# Relação entre a Relação Fibrinogênio/Albumina e a Perfusão Microvascular em Pacientes Submetidos à Intervenção Coronária Percutânea Primária para Infarto do Miocárdio com Elevação do Segmento ST: Um Estudo Prospectivo

**DOI:** 10.36660/abc.20230002

**Published:** 2023-10-16

**Authors:** Mustafa Kaplangoray, Kenan Toprak, Omer Faruk Cicek, Edhem Deveci

**Affiliations:** 1 Bilecik Şeyh Edebali University Faculty of Medicine Department of Cardiology Bilecik Turquia Bilecik Şeyh Edebali University Faculty of Medicine - Department of Cardiology, Bilecik – Turquia; 2 Harran University Faculty of Medicine Department of Cardiology Şanlıurfa Turquia Harran University Faculty of Medicine - Department of Cardiology, Şanlıurfa – Turquia; 3 University of Health Sciences Mehmet Akif İnan Research and Training Hospital Department of Cardiology Şanlıurfa Turquia University of Health Sciences Mehmet Akif İnan Research and Training Hospital - Department of Cardiology, Şanlıurfa – Turquia

**Keywords:** Infarto do Miocárdio com Elevação do segmento ST, Razão Fibrinogênio/Albumina, Imagem de Perfusão Miocárdica, Intervenção Coronária Percutânea Primária

## Abstract

**Fundamento:**

A contagem corrigida de quadros TIMI (CTFC), o grau de blush miocárdico (MBG) e a resolução do segmento ST (STR) são parâmetros utilizados para avaliar a reperfusão em nível microvascular em pacientes submetidos à intervenção coronária percutânea primária (ICPp). A relação fibrinogênio/albumina (FAR) tem sido associada a eventos trombóticos em pacientes com infarto do miocárdio com elevação do segmento ST (IAMCSST) e insuficiência venosa crônica.

**Objetivos:**

Investigar a relação do FAR com CTFC, MBG e STR.Métodos: O estudo incluiu 167 pacientes consecutivos que foram submetidos a ICPp com sucesso para IAMCSST e alcançaram fluxo TIMI-3. Os casos foram divididos em dois grupos, FAR alto (> 0,0765) e FAR baixo (≤ 0,0765), de acordo com o valor de corte desse parâmetro na análise característica do operador do receptor (ROC).  STR, CTFC e MBG foram utilizados para avaliar a reperfusão miocárdica. Valores de p<0,05 foram considerados estatisticamente significativos.

**Resultados:**

O valor CTFC, escore SYNTAX, relação neutrófilos/linfócitos, lipoproteína de baixa densidade, glicose e pico de cTnT foram significativamente maiores, enquanto STR, MBG e FEVE foram menores no grupo FAR alto. A análise de correlação de Spearman revelou relação significativa entre FAR e STR (r=-0,666, p<0,001), MBG (-0,523, p<0,001) e CTFC (r=0,731, p≤0,001). De acordo com a análise de regressão logística, FAR, glicose, pico de cTnT e dor até o tempo de Balão foram os preditores independentes mais importantes de MBG 0/1, CTFC>28 e STR<50%). A análise ROC revelou que o ponto de corte o valor de FAR≥0,0765 foi preditor de STR incompleto com sensibilidade de 71,9% e especificidade de 69,8%, MBG0/1 com sensibilidade de 72,6% e especificidade de 68,6%, e CTFC>28 com sensibilidade de 76% e uma especificidade de 65,8%.

**Conclusões:**

A FAR é um importante preditor independente de perfusão microvascular em pacientes submetidos a ICPp por IAMCSST.

**Figure:**
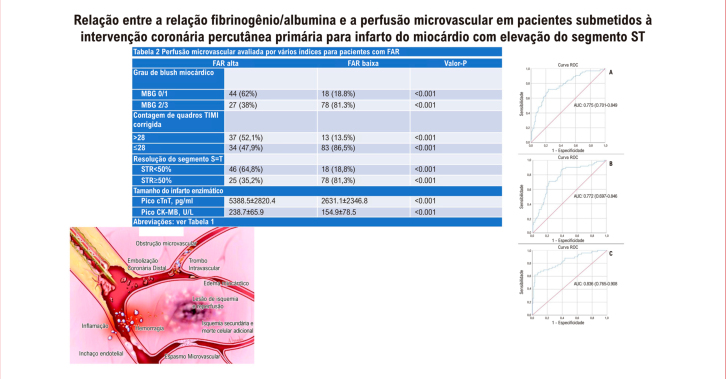
Figura Central: Relação entre a Relação Fibrinogênio/Albumina e a Perfusão Microvascular em Pacientes Submetidos à Intervenção Coronária Percutânea Primária para Infarto do Miocárdio com Elevação do Segmento ST: Um Estudo Prospectivo

## Introdução

O tratamento do infarto do miocárdio com elevação do segmento ST (IAMCSST) visa garantir a reperfusão permanente, minimizando o tempo isquêmico total.^1^ A revascularização epicárdica ideal nem sempre garante perfusão microvascular adequada.^2^ Atualmente, nenhum teste in vivo revela diretamente a circulação microvascular em humanos. No entanto, angiograficamente o grau de blush miocárdico (MBG) e trombólise corrigida no infarto do miocárdio (TIMI), contagem de quadros (CTFC) e eletrocardiograficamente, a resolução da elevação ST (STR) são frequentemente utilizados na avaliação de reperfusão microvascular em pacientes submetidos a intervenção coronária percutânea primária. (ICPp) para IAMCSST.^3^ O fibrinogênio e a albumina são dois parâmetros que desempenham um papel nas alterações hemorreológicas e na inflamação sistêmica e são, portanto, comumente usados em estudos clínicos. Estudos anteriores demonstraram que um nível elevado de fibrinogênio é um fator preditor independente de doença arterial coronariana, infarto agudo do miocárdio e risco aumentado de trombose.^4^ A albumina é uma proteína essencial do plasma humano e sabe-se que está envolvida no mecanismo de inflamação e hemostasia e inibem plaquetas.^5^ Da mesma forma, em um estudo de Kurtul et al.,^6^ a relação de não-refluxo da hipoalbuminemia foi demonstrada em pacientes submetidos a ICPp para IAMCSST. Estudos recentes também relataram que a relação fibrinogênio/albumina (FAR) fornece melhores resultados na previsão de desfechos clínicos quando comparada ao fibrinogênio ou albumina isoladamente.^7^ No presente estudo, nosso objetivo foi investigar a relação da FAR, intimamente relacionada à trombose, com STR, MBG e CTFC em pacientes submetidos a ICPp para IAMCSST.

## Métodos

### População de pacientes

**Figura 1:**
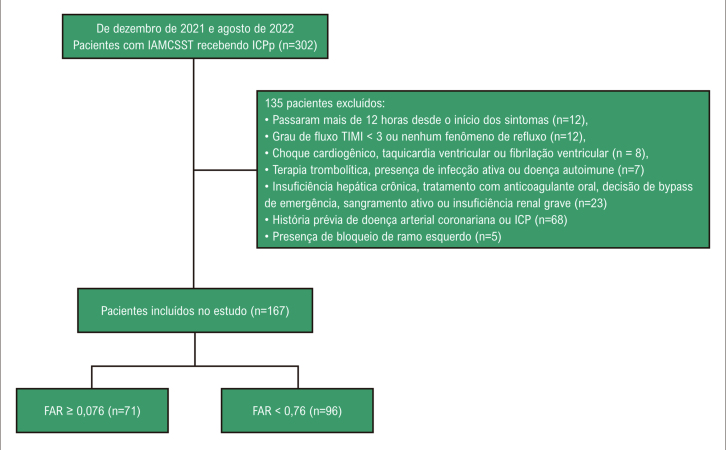
– Fluxograma do participante. IAMCSST: infarto do miocárdio com elevação do segmento ST; ICPp: intervenção coronária percutânea primária; FAR: relação fibrinogênio/albumina.

Este estudo incluiu 302 pacientes com IAMCSST que foram consecutivamente admitidos na unidade de angiografia coronária do Hospital de Treinamento e Pesquisa Sanliurfa Mehmet Akif Inan e da Universidade de Harran devido a IAMCSST entre dezembro de 2021 e agosto de 2022 e foram submetidos a ICPp com sucesso nas primeiras 12 horas após o início dos seus sintomas. O diagnóstico de IAMCSST foi feito de acordo com os critérios diagnósticos das diretrizes da Sociedade Europeia de Cardiologia (ESC).^8^ Para eliminar o efeito da estenose epicárdica residual na circulação microvascular, apenas foram considerados os casos em que fluxo TIMI-3 e estenose residual < 20%. alcançados após o procedimento estavam no estudo. Os critérios de exclusão do estudo foram: ter passado mais de 12 horas desde o início dos sintomas (n=12), grau de fluxo TIMI < 3 ou fenômeno de não refluxo após o procedimento (n=12), choque cardiogênico (n=5), taquicardia ventricular ou fibrilação ventricular (n=3), terapia trombolítica nas últimas 24 horas, presença de infecção ativa ou doença autoimune (n=7), insuficiência hepática crônica (n=3), administração oral tratamento anticoagulante (n=8), decisão de by-pass de emergência (n=4), sangramento ativo ou insuficiência renal grave (n=8), história prévia de doença arterial coronariana ou ICP (n=68) e presença de bloqueio de ramo esquerdo na eletrocardiografia (ECG) (n=5). De acordo com esses critérios, 135 pacientes foram excluídos e os 167 pacientes restantes foram incluídos neste estudo transversal prospectivo ( Figura 1 ). O protocolo do estudo foi aprovado pelo Comitê de Ética da Faculdade de Medicina da Universidade de Harran (HRU/22.8.07) e conduzido seguindo os princípios da Declaração de Helsinque. O consentimento informado por escrito foi obtido de todos os participantes.

### Análise de dados angiográficos

A angiografia foi realizada em todos os casos em múltiplas projeções pela técnica de Judkins. Antes da ICPp, a carga de 300 mg de aspirina com 600 mg de clopidogrel ou 180 mg de ticagrelor foi realizada em todos os pacientes. Imediatamente após a decisão pela intervenção coronariana, foi administrada heparina em bolus a todos os pacientes na dose de 50-70 unidades/kg. Os procedimentos angiográficos foram realizados por cardiologistas experientes, cegos para os dados e desenho do estudo. A contagem de quadros coronários foi calculada para cada paciente de acordo com o cálculo de contagem de quadros TIMI conforme descrito por Gibson et al.^9^ Como foi necessário muito tempo para a opacificação devido ao comprimento da artéria coronária descendente anterior (ADA) esquerda, o valor de CTFC calculado para ADA foi dividido por 1,7, e o CTFC foi obtido multiplicando-se o número de quadros obtidos para cada vaso por 2, considerando que os registros angiográficos foram realizados em nossa clínica como 15 quadros/segundo. Neste estudo, 28 números de quadros foram determinados como valor limite para o fluxo TIMI-3. CTFC > 28 foi considerado indicativo de distúrbio de perfusão microvascular, e CTFC ≤ 28 boa perfusão microvascular.^9 , 10^

### Análise eletrocardiográfica

O ECG padrão de 12 derivações foi realizado em todos os pacientes no momento da admissão e no final da ICPp. A avaliação do ECG foi realizada por dois cardiologistas especialistas, cegos para os demais dados dos pacientes. A elevação do segmento ST foi medida em milivolts 20 ms após o ponto J. A elevação total do segmento ST nas derivações DI, aVL e V1-V6 foi calculada para infarto não inferior, e a elevação total do segmento ST nas derivações D2, D3, aVF, V5 e V6 para infarto inferior. A depressão total na elevação do ST determinada na localização especificada no final da ICPp foi dividida pela elevação total do ST inicial para obter a resolução do ST (STR). De acordo com esse parâmetro, os casos foram classificados como STR completo (≥ 50%), STR incompleto (<50) e com STR < 50% indicando distúrbio circulatório microvascular.^11^

### Grau de blush miocárdica

MBG é uma medida da opacificação miocárdica pelo meio de contraste fornecido pela artéria responsável pelo infarto pós-reperfusão. O cálculo do MBG de todos os pacientes foi realizado conforme descrito anteriormente por van’t Hof et al.^12^ De acordo com a avaliação do MBG, os pacientes foram classificados em grau 0 (sem blush miocárdico), grau 1 (blush miocárdico mínimo ou intensidade de contraste), grau 2 (blush ou intensidade miocárdica moderada, mas menos blush do que o blush miocárdico ipsilateral ou contralateral não artéria associada infectada durante a angiografia) e grau 3 (coração miocárdico normal ou intensidade de contraste). Considerou-se que MBG 0/1 indicava obstrução microvascular, enquanto MBG graus 2 e 3 foram aceitos como boa perfusão microvascular.^13^

### Medições laboratoriais

As amostras de sangue de todos os participantes foram coletadas da região antecubital no momento da admissão no hospital. A FAR foi calculada como a razão entre o nível sérico de fibrinogênio e o nível de albumina na admissão. O nível plasmático de fibrinogênio foi medido pelo método de coagulação utilizando o analisador automático de coagulação STA CompactMax. Os níveis de albumina, marcadores de dano miocárdico convencionais troponina T e creatina quinase-MB (CK-MB) e outros parâmetros bioquímicos de rotina também foram medidos a partir de amostras de sangue coletadas na admissão usando o analisador automático Abbott Architect C16000.

### Tamanho do infarto enzimático

A troponina T e a CK-MB foram medidas nas horas 0, 6, 12, 18, 24, 36, 48 e 72 após ICPp. Após o procedimento, a extensão do tamanho do infarto foi avaliada utilizando pico de troponina T cardíaca (cTnT) e CK-MB.

### Função ventricular esquerda

Um exame ecocardiográfico foi realizado em todos os pacientes nas primeiras 24 horas após a ICPp, conforme recomendado pelas diretrizes da ESC/American Heart Association. O método de Simpson modificado foi utilizado para a fração de ejeção do ventrículo esquerdo (FEVE).

### Análise estatística

As análises estatísticas dos dados coletados da pesquisa foram realizadas por meio do pacote de software Statistical Package for the Social Sciences (SPSS for Windows, versão 22.0, IBM Corp., Armonk, NY, US, 2016). As variáveis contínuas com distribuição normal foram descritas como média ± desvio padrão, e as variáveis contínuas sem distribuição normal foram descritas como mediana e intervalo interquartil.

As variáveis categóricas foram expressas em porcentagens e comparadas com o teste qui-quadrado ou exato de Fisher. A normalidade dos dados foi verificada por meio do teste de Kolmogorov-Smirnov. Dois grupos foram comparados com o teste t de amostras independentes para dados contínuos em conformidade com a distribuição normal. Os dados com distribuição não normal foram comparados com o teste U de Mann-Whitney. A relação entre os parâmetros foi determinada pelo coeficiente de correlação de Spearman. A análise da característica de operação do receptor (ROC) foi utilizada para obter o valor de corte do FAR para a predição de STR (0,0738), MBG (0,0788) e CTFC (0,0769). O valor de corte para FAR alto e baixo foi determinado pela média desses três valores (0,0765). As análises de regressão logística univariada e multivariada foram utilizadas para identificar os preditores independentes de STR incompleto, MBG 0/1 e CTFC > 28. P < 0,05 foi considerado estatisticamente significativo.

## Resultados

O estudo incluiu 167 pacientes com idade média de 59,4 ± 11,1 anos. Noventa e seis (57,5%) pacientes eram do sexo masculino. As características demográficas e os dados clínicos basais dos pacientes são apresentados na Tabela 1 . Os pacientes incluídos no estudo foram divididos em grupos de alta e baixa FAR de acordo com o valor de corte desse parâmetro na análise ROC (0,0765). Quando os resultados laboratoriais foram comparados entre os dois grupos, foi determinado que o grupo FAR alta apresentou valores significativamente mais elevados de escore SYNTAX, idade, relação neutrófilos/linfócitos (NLR), classe Killip ≥ 2, glicose, lipoproteína de baixa densidade (LDL), pico de TnT e taxa de diabetes ( Tabela 1 ).

**Tabela 1:** – Relação entre características clínicas e a FAR em pacientes com IAMCSST submetidos à ICPp

Característica	FAR Alta (n=71)	FAR Baixo (n=96)	p
**Idade, anos**	61,4 ±10,6	57,8±11,2	0,043
**Homens, n (%)**	40 (56,3%)	56 (58,3%)	0,797
**IMC, kg/m** ^**2**^	28,2±3,8	27,3±3,4	0,105
**Tabagismo, n (%)**	29 (40,8%)	38 (39,6%)	0,869
**Hipertensão, n (%)**	26 (36,6%)	23 (24,0%)	0,076
**Diabetes, n (%)**	35 (49,3%)	32 (33,3%)	0,037
**PAS, mmHg**	128,3±18,7	126,6±18,9	0,207
**PAD, mmHg**	80,9±13,4	81,2±12,6	0,917
**Frequência cardíaca, /min**	77,7±12,6	75,1±13,9	0,212
**Tempo dor-balão (min)**	70,6±20,1	64,5±23,8	0,076
**Escore SYNTAX**	19,4±6,2	16,9±7,3	0,004
**FEVE, %**	41,8±6,5	46,4±6,0	<0,001
**Classe Killip**			
Classe 1, n	54 (76,1%)	88 (91,7%)	0,005
Classe ≥2, n	17 (23,9%)	8 (8,3%)	0,005
**Histórico médico**			
Ácido acetilsalicílico	23 (31,5%)	23 (24,5%)	0,313
Estatina	15 (20,5%)	19 (20,2%)	0,957
Betabloqueador	23 (31,5%)	20 (21,3%)	0,134
IECA/BRA	27 (37,0%)	25 (26,6%)	0,150
**Achados laboratoriais**			
Contagem glóbulos brancos, ( x10^3^/ µL)	12,3 (10,1-14,9)	12,0 (10,9-13,9)	0,965
Razão neutrófilos/linfócitos	2,9 (2,1-3,7)	1,9 (1,3-2,9)	< 0,001
Hemoglobina, g/l	13,9 (12,5-15,1)	14,2 (13-15,3)	0,495
Contagem de plaquetas, ( x10^3^/ µL)	256 (164-354)	247 (198-319)	0,245
Glicose	172 (145-201)	135 (122-175)	<0,001
ALT, UI/l	35,7±21,2	36,1±27,7	0,938
AST, UI/l	29,7±18,8	28,5±16,2	0,658
Creatinina, μmol/l	0,95±0,25	0,94±0,26	0,759
Nitrogênio ureico no sangue, mmol/l	39,3±11,7	35,7±12,2	0,054
Colesterol total, mmol/l	199 (185-214)	196 (178-205)	0,149
HDL, mmol/l	35 (33-41)	37 (33-41)	0,214
LDL, mmol/l	152 (136-161)	139 (122-150)	<0,001
Triglicerídeo, mmol/l	178 (156-199)	177 (155-197)	0,461
**Artéria relacionada ao infarto**			
ADA, n (%)	32 (45,1%)	43 (44,8%)	0,609
CX, n (%)	18 (25,4)	19 (21,3)	
CD, n (%)	21 (29,6)	34 (35,4)	

*FAR: Razão Fibrinogênio-Albumina; IMC: índice de massa corporal; PAS: pressão arterial sistólica; PAD: pressão arterial diastólica; SYNTAX: Sinergia entre intervenção coronária percutânea com TAXus; LDL-C: colesterol de lipoproteína de baixa densidade; ALT: alanina aminotransferase; AST: aspartato aminotransferase; HDL-C: colesterol de lipoproteína de alta densidade; IECA: inibidor da enzima conversora de angiotensina; BRA: bloqueador do receptor de angiotensina; CCB: bloqueadores dos canais de cálcio; STR: Resolução do Segmento ST; ADA: artéria descendente anterior esquerda; CX: artéria circunflexa esquerda; CD: artéria coronária direita; IRA: artéria relacionada ao infarto; FEVE: fração de ejeção do ventrículo esquerdo.*

**Figura 2:**
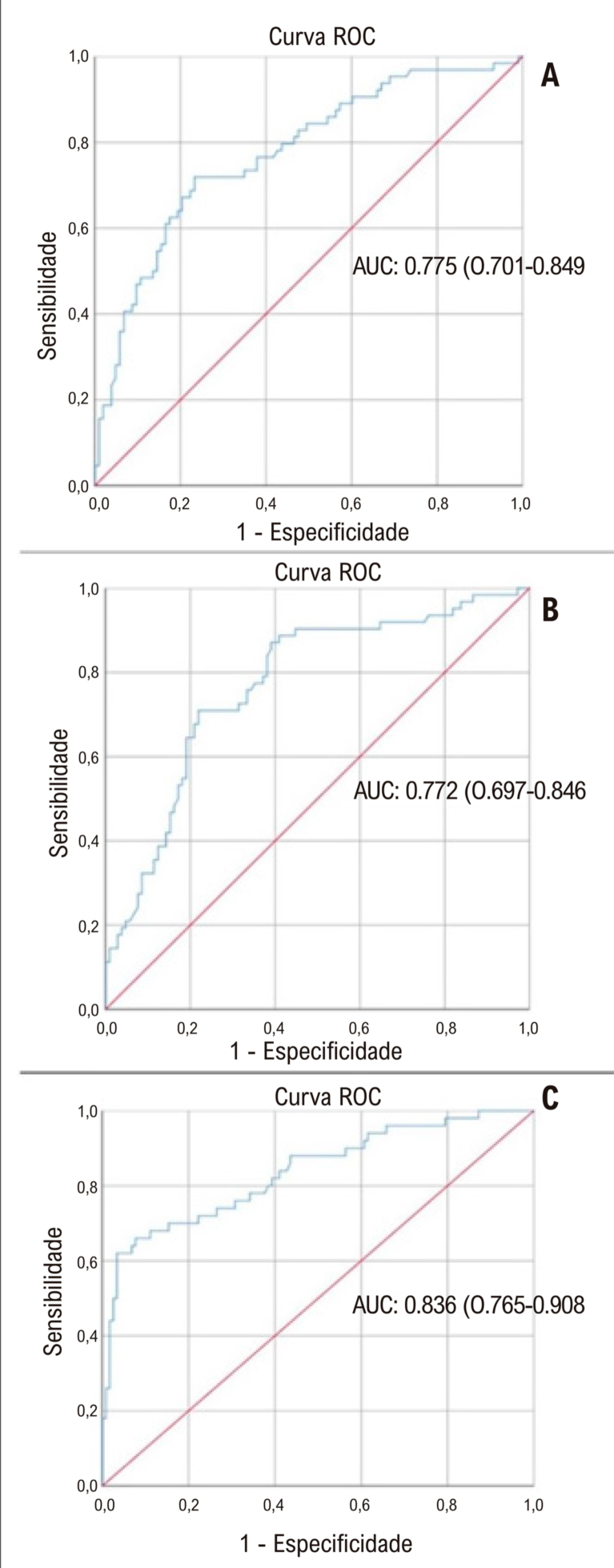
– Análise da curva característica de operação do receptor dos níveis de FAR para predição de resolução incompleta do segmento ST (A), blush miocárdico grau 0/1 (B) e contagem corrigida de quadros TIMI >28 (C). AUC: área sob a curva; FAR: relação fibrinogênio/albumina.

A FEVE foi menor no grupo FAR alta ( Tabela 2 ). Não houve diferença significativa entre os grupos de FAR alta e baixa em relação ao histórico de tratamento médico e às artérias coronárias associadas ao infarto do miocárdio. As taxas de pacientes com MBG2/3, STR ≥ 50% e CTFC ≤ 28 foram significativamente maiores no grupo FAR baixa (p < 0,001 para todos) ( Tabela 2 ). De acordo com a análise de correlação, a FAR foi positivamente correlacionada com pico de cTnT, glicose, escore SYNTAX, NLR, CTFC e LDL e negativamente correlacionada com STR, FEVE e MBG. A FAR teve a correlação positiva mais forte com o CTFC e a correlação negativa mais forte com o STR ( Tabela 3 ). A análise de regressão logística revelou que a FAR foi o preditor independente mais importante de MBG 0/1, CTFC > 28 e STR incompleto ( Tabela 4 ). Na análise ROC, quando o valor de corte do FAR foi considerado ≥ 0,0765 (área sob a curva: 0,775, IC: 0,701-0,849), previu STR incompleto com sensibilidade de 71,9% e especificidade de 68,9%, MBG0/1 com sensibilidade de 72,6% e especificidade de 68,6%, CTFC > 28 com sensibilidade de 76% e especificidade de 65,8% ( Figura 2 ).

**Tabela 2:** – Perfusão microvascular avaliada por vários índices para pacientes com FAR

	FAR Alta	FAR Baixa	Valor p
**Grau de blush miocárdico**			
MBG 0/1	44 (62%)	18 (18,8%)	<0,001
MBG 2/3	27 (38%)	78 (81,3)	<0,001
**Contagem de quadros TIMI corrigida**			
>28	37 (52,1%)	13 (13,5%)	<0,001
≤28	34 (47,9%)	83 (86,5%)	<0,001
**Resolução do segmento ST**			
STR<50%	46 (64,8%)	18 (18,8%)	<0,001
STR≥50%	25 (35,2%)	78 (81,3%)	<0,001
**Tamanho do infarto enzimático**			
Pico de cTnT, pg/ml	5100 (3410-7890)	1670 (1022-4092)	<0,001
Pico CK-MB, U/L	255 (201-300)	133 (98-229)	<0,001

*FAR: Razão Fibrinogênio-Albumina; STR: Resolução do Segmento ST; CTFC: contagem corrigida de quadros TIMI; MBG: grau de blush miocárdico*

**Tabela 3:** – Correlação entre FAR e parâmetros clínicos, laboratoriais e angiográficos

Variáveis	FAR	
	r	p
NLR	0,389	<0,001
STR	-0,666	<0,001
Escore Syntax	0,223	0,004
FEVE	-0,398	<0,001
Nível pico de Troponina	0,582	<0,001
CTFC	0,731	<0,001
MBG	-0,523	<0,001
Tempo dor-balão (min)	0,158	0,041
LDL-C	0,245	<0,001
Glicose	0,387	<0,001

*FAR: Razão Fibrinogênio-Albumina; LDL-C: colesterol de lipoproteína de baixa densidade; FEVE: fração de ejeção do ventrículo esquerdo; CTFC: contagem corrigida de quadros TIMI; MBG: grau de blush miocárdico; STR: Resolução do Segmento ST; NLR: relação neutrófilos/linfócitos.*

**Tabela 4:** – Regressão Logística Identificando Fatores de Risco para MBG 0/1, CTFC>28 e STR<50%

Variáveis	MBG 0/1		CTFC>28		STR<50%	
	OR ajustado (IC 95%)	Valor p	OR ajustado (IC 95%)	Valor p	OR ajustado (IC 95%)	Valor p
FAR	0,219 (0,090-0,531)	<0,001	0,230(0,093-0,567)	<0,001	0,097 (0,037-0,250)	<0,001
Idade	0,970 (0,931-1,10)	0,143	0,089(0,949-1,030)	0,586	1,006 (0,970-1,044)	0,750
DM	0,982(0,411-2,346)	0,967	1,448 (0,587-3,573)	0,422	0,867 (0,375-2,002)	0,738
SYNTAX	1,046 (0,978-1,119)	0,190	0,909 (0,854-0,967)	0,002	0,977 (0,925-1,032)	0,403
NLR	0,710(0,520-0,970)	0,031	0,989 (0,875-1,119)	0,864	0,929 (0,758-1,138)	0,476
Glicose	1,010 (0,989-1,022)	0,023	0,991 (0,982-1,001)	0,048	1.013(1.002-1.024)	0,018
LDL-C	1,007(0,988-1,027)	0,463	0,999 (0,981-1,017)	0,900	1,001(0,985-1,018)	0,881
Pico de cTnT	1,010(0,0997-1,028)	0,041	1,000 (0,988-1,012)	<0,001	1.001 (1.000-1.002)	<0,001
Tempo da dor ao balão	1,001(0,982-1,020)	0,025	1,035 (1,024-1,046)	0,002	0,977(0,960-0,994)	0,007

*FAR: Razão Fibrinogênio-Albumina; LDL-C: colesterol de lipoproteína de baixa densidade; CTFC: contagem corrigida de quadros TIMI; MBG: grau de blush miocárdico; STR: Resolução do Segmento ST; NLR: relação neutrófilos/linfócitos.*

## Discussão

Este estudo investigou o efeito da FAR nos marcadores de reperfusão microvascular MBG, CTF e STR. Até onde sabemos, este é o primeiro estudo prospectivo realizado para esse fim na literatura. Nossos resultados mostraram que o aumento da FAR foi associado com baixa MBG, diminuição da STR e alta CTFC. Também foi revelado que a FAR foi o fator independente mais importante na previsão desses parâmetros.

A STR após ICPp angiograficamente bem-sucedida está intimamente relacionada ao dano tecidual e à perfusão, e muitos estudos demonstraram que a STR reduzida está associada a resultados clínicos ruins.^14 , 15^ No presente estudo, também foi observado que todos os pacientes com STR incompleta estavam em o grupo FAR alta. Também foi demonstrado que um nível alto de FAR estava associado ao aumento do pico de cTnT e aos baixos valores de FEVE, que são marcadores de dano tecidual. Níveis aumentados de fibrinogênio causam aumento na ativação e agregação plaquetária, resultando em um estado de hipercoagulabilidade. Além disso, a estrutura de um coágulo de fibrina está intimamente relacionada com o nível de fibrinogênio. Foi relatado que a fibrina formada in vitro é mais densa e mais resistente à fibrinólise em altas concentrações de fibrinogênio do que a fibrina formada em baixas concentrações.^16^ A albumina, o outro componente da FAR, é a principal proteína do soro humano e inibe a agregação plaquetária aumentando produção de prostaglandina D2. Além disso, sabe-se que um nível baixo de albumina causa um aumento na viscosidade do sangue e deterioração da função endotelial. Além disso, sabe-se que a albumina tem efeito antioxidante e relação inversa com a inflamação.^7^ À luz destes mecanismos, consideramos que um valor elevado de FAR pode causar uma diminuição na STR ao interromper a reperfusão a nível microvascular.

Em estudos recentes, o escore CTFC tem sido usado para avaliar a reperfusão após ICP, em vez do escore de fluxo TIMI, uma vez que este último oferece uma avaliação mais objetiva e quantitativa.^17 , 18^ Muitos estudos demonstraram que as medidas de CTFC fornecem resultados úteis na previsão de desfechos clínicos. Sabe-se que o fluxo coronário acelerado está associado a bons resultados clínicos. Foi demonstrado que um CTFC baixo após a reperfusão está associado a uma baixa taxa de mortalidade.^19^ No presente estudo, utilizamos o CTFC para avaliar a reperfusão miocárdica fornecida pela artéria responsável pelo infarto do miocárdio e determinamos que o aumento da FAR foi o preditor mais importante de CTFC. > 28. Considera-se que os mecanismos responsáveis pelo distúrbio circulatório microvascular são macroembolização distal, microembolização, formação de trombo local na região distal, liberação de radicais de oxigênio no ambiente, aumento do nível de cálcio nos miócitos, edema intersticial, disfunção endotelial, vasoconstrição e inflamação.^20^ Muitos estudos demonstraram a relação da FAR com os fatores acima mencionados.^21^ Da mesma forma, em nosso estudo, a circulação microvascular prejudicada em pacientes com FAR elevada pode ter resultado em uma CTFC mais elevada neste grupo de pacientes.

O sucesso angiográfico após ICP é definido como uma redução de pelo menos 50% na estenose com a obtenção de fluxo TIMI de grau 3 após a dilatação do balão e um máximo de 10% de estenose residual após implante de stent.^12^ No entanto, resultados angiográficos bem-sucedidos nem sempre significam reperfusão miocárdica bem-sucedida. Devido à sua praticidade e facilidade de uso, o MBG é frequentemente utilizado na prática clínica para determinar a reperfusão em nível microvascular. MBG 0/1 é definido como um distúrbio de perfusão no nível microvascular.^13^ Uma metanálise recente de 8.044 pacientes mostrou que MBG 0/1 após angioplastia primária estava associada à mortalidade por todas as causas.^22^ No presente estudo, determinamos que o aumento do FAR foi um importante fator preditor para MBG 0/1. O aumento do efeito trombogênico do fibrinogênio isolado e o baixo nível de albumina aumentam a agregação plaquetária, levando a distúrbios de perfusão no nível do tecido. Além disso, considera-se que a FAR está relacionada ao aumento da viscosidade sanguínea e à trombogenicidade.^7^ Tem sido sugerido que o aumento da carga trombótica desses fatores prejudica a perfusão miocárdica, causando microembolismo distal.^23 - 25^ Paralelamente a esses mecanismos, um valor elevado de FAR foi associado ao MBG 0/1 em nosso estudo. Outro achado importante do nosso estudo é que os pacientes do grupo FAR alta tiveram um escore SYNTAX mais alto, o que é consistente com os resultados relatados por Erdoğan et al.^26^ Em nosso estudo, também descobrimos que os níveis de glicose, LDL-C e a NLR foram significativamente maiores no grupo FAR alta do que no grupo FAR baixa. Recentemente, a FAR tem sido relatada como um marcador de inflamação relacionado a diversas doenças, incluindo diabetes mellitus e hiperlipidemia.^27 , 28^

### Limitações

Houve algumas limitações neste estudo devido ao seu desenho. Primeiro, a população do estudo era relativamente pequena. Segundo, medimos apenas na admissão e não avaliamos após a fase aguda, o que pode ser considerado uma limitação importante. Terceiro, a falta de efeito deste parâmetro sobre os resultados cardiovasculares, incluindo reintervenção e mortalidade.

## Conclusões

Concluindo, com base em nossos resultados, consideramos que a FAR é um marcador de fácil acesso e baixo custo que pode ser utilizado pelos médicos na avaliação da perfusão microvascular em pacientes submetidos à ICPp para IAMCSST. Além disso, este marcador pode ser usado para determinar opções de tratamento adjuvante para ICPp.
