# Supplementary material for: Relação entre a Relação Fibrinogênio/Albumina e a Perfusão Microvascular em Pacientes Submetidos à Intervenção Coronária Percutânea Primária para Infarto do Miocárdio com Elevação do Segmento ST: Um Estudo Prospectivo
Source: Arq Bras Cardiol. 2023 Oct 16;120(11):e20230002. [Article in Portuguese] doi: 10.36660/abc.20230002 (PMC12087635; doi:10.36660/abc.20230002)
Supplement: Translation

# Translation: Relationship between the Fibrinogen/Albumin Ratio and Microvascular Perfusion in Patients Undergoing Primary Percutaneous Coronary Intervention for ST-Elevated Myocardial Infarction: A Prospective Study

**Authors:** Mustafa Kaplangoray, Kenan Toprak, Omer Faruk Cicek, Edhem Deveci

**Affiliations:** 1 Bilecik Şeyh Edebali University Faculty of Medicine Department of Cardiology Bilecik Turquia Bilecik Şeyh Edebali University Faculty of Medicine - Department of Cardiology, Bilecik – Turquia; 2 Harran University Faculty of Medicine Department of Cardiology Şanlıurfa Turquia Harran University Faculty of Medicine - Department of Cardiology, Şanlıurfa – Turquia; 3 University of Health Sciences Mehmet Akif İnan Research and Training Hospital Department of Cardiology Şanlıurfa Turquia University of Health Sciences Mehmet Akif İnan Research and Training Hospital - Department of Cardiology, Şanlıurfa – Turquia

**Keywords:** ST-Elevation Myocardial Infarction, Fbrinogen-to-Albumin Ratio, Myocardial Perfusion Imaging, Primary Percutaneous Coronary Intervention

## Abstract

**Background:**

Correct TIMI frame count (CTFC), myocardial blush grade (MBG), and ST-segment resolution (STR) are parameters used to evaluate reperfusion at the microvascular level in patients that have undergone primary percutaneous coronary intervention (pPCI). Fibrinogen-to-albumin ratio (FAR) has been associated with thrombotic events in patients with ST-elevation myocardial infarction (STEMI) and chronic venous insufficiency.

**Objectives:**

To investigate the relationship of FAR with CTFC, MBG, and STR. Methods: The study included 167 consecutive patients who underwent successful pPCI for STEMI and achieved TIMI-3 flow. The cases were divided into two groups, high (>0.0765) and low FAR (≤0.0765), according to the cut-off value of this parameter in the receiver operator characteristic analysis (ROC). STR, CTFC, and MBG were used to evaluate myocardial reperfusion. P values<0.05 were considered statistically significant.

**Results:**

CTFC value, SYNTAX score, neutrophil/lymphocyte ratio, low-density lipoprotein, glucose, and peak cTnT were significantly higher, whereas STR, MBG, and LVEF were lower in the high FAR group. Spearman’s correlation analysis revealed a significant relationship between the FAR and STR (r=-0.666, p<0.001), MBG (-0.523, p<0.001), and CTFC (r=0.731, p≤0.001). According to the logistic regression analysis, FAR, glucose, peak cTnT, and pain to balloon time were the most important independent predictors of MBG 0/1, CTFC>28, and STR<50%).ROC analysis revealed that the cut-off value of FAR≥0.0765 was a predictor of incomplete STR with a sensitivity of 71.9 % and a specificity of 69.8 %, MBG0/1 with a sensitivity of 72.6 % and a specificity of 68.6 %, and CTFC >28 with a sensitivity of 76 % and a specificity of 65.8 %.

**Conclusions:**

FAR is an important independent predictor of microvascular perfusion in patients undergoing pPCI for STEMI.

**Central Illustration:** : Relationship between the Fibrinogen/Albumin Ratio and Microvascular Perfusion in Patients Undergoing Primary Percutaneous Coronary Intervention for ST-Elevated Myocardial Infarction: A Prospective Study

## Introduction

ST-segment elevation myocardial infarction (STEMI) treatment aims to ensure permanent reperfusion by minimizing the total ischemic time.^1^Optimal epicardial revascularization does not always guarantee adequate microvascular perfusion.^2^Currently, no in vivo test directly reveals microvascular circulation in humans. However, angiographically myocardial blush grade (MBG) and correct thrombolysis in myocardial infarction (TIMI) frame count (CTFC) and electrocardiographically ST-elevation resolution (STR) are frequently used in the evaluation of microvascular reperfusion in patients who have undergone primary percutaneous coronary intervention (pPCI) for STEMI.^3^Fibrinogen and albumin are two parameters that play a role in hemorheological changes and systemic inflammation and are, therefore, commonly used in clinical studies. Previous studies have shown that a high fibrinogen level is an independent predictor factor of coronary artery disease, acute myocardial infarction, and an increased risk of thrombosis.^4^Albumin is an essential protein of human plasma, and it is known to be involved in the mechanism of inflammation and hemostasis and inhibit platelets.^5^Similarly, in a study by Kurtul et al.,^6^the no-reflow relationship of hypoalbuminemia was shown in patients who underwent pPCI for STEMI. Recent studies have also reported that the fibrinogen/albumin ratio (FAR) provides better results in predicting clinical outcomes when compared to fibrinogen or albumin alone.^7^In the current study, we aimed to investigate the relationship of FAR, closely related to thrombosis, with STR, MBG, and CTFC in patients that had undergone pPCI for STEMI.

## Materials and methods

### Patient population

This study included 302 STEMI patients who were consecutively admitted to the coronary angiography unit of Sanliurfa Mehmet Akif Inan Training and Research Hospital and Harran University due to STEMI between December 2021 and August 2022 and underwent successful pPCI within the first 12 hours of the onset of their symptoms. The diagnosis of STEMI was made according to the diagnostic criteria of the European Society of Cardiology (ESC) guidelines.^8^To eliminate the effect of residual epicardial stenosis on microvascular circulation, only cases in which TIMI-3 flow and residual stenosis < 20% were achieved after the procedure were in the study. The exclusion criteria of the study were as follows: more than 12 hours have passed since the onset of symptoms (n = 12), TIMI flow grade < 3 or no-reflow phenomenon after the procedure (n = 12), cardiogenic shock (n = 5), ventricular tachycardia or ventricular fibrillation (n = 3), thrombolytic therapy within the last 24 hours, presence of active infection or autoimmune disease (n = 7), chronic liver failure (n = 3), oral anticoagulant treatment (n = 8), emergency bypass decision (n = 4), active bleeding or severe renal failure (n = 8), previous history of coronary artery disease or PCI (n = 68), and presence of left bundle branch block on electrocardiography (ECG) (n = 5). According to these criteria, 135 patients were excluded, and the remaining 167 patients were included in this prospective cross-sectional study ( Figure 1 ). The study protocol was approved by the Ethics Committee of Harran University Faculty of Medicine (HRU/22.8.07) and conducted following the tenets of the Declaration of Helsinki. Written informed consent was obtained from all the participants.

**Figure 1:** – *The participant flowchart.*

### Angiographic data analysis

Angiography was performed in all the cases in multiple projections using the Judkins technique. Prior to pPCI, the loading of 300 mg aspirin with 600 mg of clopidogrel or 180 mg of ticagrelor was performed in all the patients. Immediately after the decision for coronary intervention was made, bolus heparin was administered to all the patients at a dose of 50-70 units/kg. Angiographic procedures were performed by experienced cardiologists blinded to the study data and design. The coronary frame count was calculated for each patient according to the TIMI frame count calculation as described by Gibson et al.^9^Since a long time was taken for opacification due to the left anterior descending coronary artery (LAD) length, the TFC value calculated for LAD was divided by 1.7, and CTFC was obtained by multiplying the number of frames obtained for each vessel by 2, considering that angiographic recordings were taken in our clinic as 15 frames/second. In this study, 28 frame numbers were determined as the threshold value for TIMI-3 flow. CTFC > 28 was considered to indicate microvascular perfusion disorder, and CTFC ≤ 28 good microvascular perfusion.^9 , 10^

### Electrocardiographic analysis

The 12-lead standard ECG was performed in all the patients at the time of admission and end of the pPCI. The ECG evaluation was performed by two expert cardiologists blinded to the remaining data of the patients. ST-segment elevation was measured in millivolts 20 ms after the J point. Total ST-segment elevation in leads DI, aVL, and V1-V6 was calculated for non-inferior infarction, and total ST-segment elevation in leads D2, D3, aVF, V5, and V6 for inferior infarction. Total depression in ST elevation determined at the specified localization at the end of pPCI was divided by the initial total ST elevation to obtain ST resolution (STR). According to this parameter, the cases were classified as complete STR (≥ 50%), incomplete STR (<50), and with STR < 50 % indicating microvascular circulatory disorder.^11^

### Myocardial blush grade

MBG is a measure of myocardial opacification by contrast medium supplied by the artery responsible for post-reperfusion infarction. The MBG calculation of all the patients was performed as previously described by van’t Hof et al.^12^According to the MBG evaluation, the patients were classified to have grade 0 (no myocardial blushing), grade 1 (minimal myocardial blush or contrast intensity), grade 2 (moderate myocardial blush or intensity but less blush than the ipsilateral or contralateral non-infected associated artery during angiography), and grade 3 (normal myocardial blush or contrast intensity). MBG 0/1 was considered to indicate microvascular obstruction, while MBG grades 2 and 3 were accepted as good microvascular perfusion.^13^

### Laboratory measurements

The blood samples of all the participants were taken from the antecubital region at the time of admission to the hospital. FAR was calculated as the ratio of the serum fibrinogen level to the albumin level at admission. The plasma fibrinogen level was measured with the coagulation method using the STA CompactMax automatic coagulation analyzer. The levels of albumin, myocardial damage markers conventional troponin T and creatine kinase-MB (CK-MB), and other routine biochemical parameters were also measured from the blood samples taken at admission using the Abbott Architect C16000 autoanalyzer.

### Enzymatic infarct size

Troponin T and CK-MB were measured at hours 0, 6, 12, 18, 24, 36, 48, and 72 after pPCI. After the procedure, the extent of infarct size was evaluated using peak cardiac troponin T (cTnT) and CK-MB.

### Left ventricular function

An echocardiographic examination was performed in all patients within the first 24 hours following the pPCI as the ESC/American Heart Association guidelines recommended. Modified Simpson’s method was used for left ventricular ejection fraction (LVEF).

### Statistical analysis

The statistical analyses of the collected research data were carried out using the Statistical Package for the Social Sciences (SPSS for Windows, version 22.0, IBM Corp., Armonk, NY, U.S., 2016) software package. Continuous variables with normal distribution were described as mean ± standard deviation, and continuous variables without normal distribution were described as median and interquartile range.

Categorical variables were expressed as percentages and compared with the chi-square or Fisher’s exact test. Data normality was verified using the Kolmogorov-Smirnov test. Two groups were compared with the independent-samples t-test for continuous data conforming to the normal distribution. Non-normally distributed data were compared with the Mann-Whitney U test. The relationship between parameters was determined using Spearman’s correlation coefficient. The receiver-operating characteristic (ROC) analysis was used to obtain the cut-off value of FAR for the prediction of STR (0.0738), MBG (0.0788), and CTFC (0.0769). The cut-off value for high and low FAR was determined by averaging these three values (0.0765). The univariate and multivariate logistic regression analyses were used to identify the independent predictors of incomplete STR, MBG 0/1, and CTFC > 28. P < 0.05 was considered statistically significant.

## Results

The study included 167 patients with a mean age of 59.4 ± 11.1 years. Ninety-six (57.5%) patients were male. The demographic characteristics and baseline clinical data of the patients are shown in Table 1 . The patients included in the study were divided into high and low FAR groups according to the cut-off value of this parameter in the ROC analysis (0.0765). When the laboratory results were compared between the two groups, it was determined that the high FAR group had significantly higher values of SYNTAX score, age, neutrophil/lymphocyte ratio (NLR), Killip class ≥ 2, glucose, low-density lipoprotein (LDL), peak TnT and diabetes rate ( Table 1 ).

**Table 1:** – Relationship between clinical characteristics and the FAR in patients with STEMI undergoing pPCI

Characteristic	High FAR (n=71)	Low FAR (n=96)	p
**Age, years**	61.4 ±10.6	57.8±11.2	0.043
**Males, n (%)**	40 (56.3%)	56 (58.3%)	0.797
**BMI, kg/m** ^**2**^	28.2±3.8	27.3±3.4	0.105
**Smoking, n (%)**	29 (40.8%)	38 (39.6%)	0.869
**Hypertension, n (%)**	26 (36.6%)	23 (24.0%)	0.076
**Diabetes, n (%)**	35 (49.3%)	32 (33.3%)	0.037
**SBP, mmHg**	128.3±18.7	126.6±18.9	0.207
**DBP, mmHg**	80.9±13.4	81.2±12.6	0.917
**Heart rate, /min**	77.7±12.6	75.1±13.9	0.212
**Pain-to-balloon time (min)**	70.6±20.1	64.5±23.8	0.076
**SYNTAX score**	19.4±6.2	16.9±7.3	0.004
**LVEF,%**	41.8±6.5	46.4±6.0	<0.001
**Killip class**			
Class 1, n	54 (76.1%)	88 (91.7%)	0.005
Class ≥2, n	17 (23.9%)	8 (8.3%)	0.005
**Medical history**			
Acetylsalicylic acid	23 (31.5%)	23 (24.5%)	0.313
Statin	15 (20.5%)	19 (20.2%)	0.957
Beta-blocker	23 (31.5%)	20 (21.3%)	0.134
ACEI/ARB	27 (37.0%)	25 (26.6%)	0.150
**Laboratory findings**			
WBC, ( x10^3^/ µL)	12.3 (10.1-14.9)	12.0 (10.9-13.9)	0.965
Neutrophil/Lymphocyte ratio	2.9 (2.1-3.7)	1.9 (1.3-2.9)	< 0.001
Hemoglobin, g/l	13.9 (12.5-15.1)	14.2 (13-15.3)	0.495
Platelet count, ( x10^3^/ µL)	256 (164-354)	247 (198-319)	0.245
Glucose	172 (145-201)	135 (122-175)	<0.001
ALT, IU/l	35.7±21.2	36.1±27.7	0.938
AST, IU/l	29.7±18.8	28.5±16.2	0.658
Creatinine, μmol/l	0.95±0.25	0.94±0.26	0.759
Blood urea nitrogen, mmol/l	39.3±11.7	35.7±12.2	0.054
Total cholesterol, mmol/l	199 (185-214)	196 (178-205)	0.149
HDL, mmol/l	35 (33-41)	37 (33-41)	0.214
LDL, mmol/l	152 (136-161)	139 (122-150)	<0.001
Triglyceride, mmol/l	178 (156-199)	177 (155-197)	0.461
**İnfarct-related artery**			
LAD, n (%)	32 (45.1%)	43 (44.8%)	0.609
CX,n (%)	18 (25.4)	19 (21.3)	
RCA, n (%)	21 (29.6)	34 (35.4)	

*FAR: Fibrinogen-Albumin Ratio; BMI: body mass index; SBP: systolic blood pressure; DBP: diastolic blood pressure; SYNTAX: SYNergy between percutaneous coronary interventin with TAXus; LDL-C: low-density lipoprotein cholesterol; ALT: Alanine Aminotransferase; AST: Aspartate Aminotransferase; HDL-C: high density lipoprotein cholesterol; ACEI: angiotensin-converting enzyme inhibitor; ARB: angiotensin receptor blocker; CCB: calcium channel blockers; STR: ST-Segment Resolution; LAD: left anterior descending artery; CX: left circumflex artery; RCA: right coronary artery artery; IRA: infarct-related artery; LVEF: left ventricular ejection fraction.*

LVEF was lower in the high FAR group ( Table 2 ). There was no significant difference between the high and low FAR groups regarding medical treatment history and coronary arteries associated with myocardial infarction. The rates of patients with MBG2/3, STR ≥ 50 %, and CTFC ≤ 28 were significantly higher in the low FAR group (p < 0.001 for all) ( Table 2 ). According to the correlation analysis, FAR was positively correlated with peak cTnT, glucose, SYNTAX score, NLR, CTFC, and LDL and negatively correlated with STR, LVEF, and MBG. FAR had the strongest positive correlation with CTFC and the strongest negative correlation with STR ( Table 3 ). The logistic regression analysis revealed that FAR was the most important independent predictor of MBG 0/1, CTFC > 28, and incomplete STR ( Table 4 ). In the ROC analysis, when the cut-off value of FAR was taken as ≥ 0.0765 (area under the curve: 0.775, CI: 0.701-0.849), it predicted incomplete STR with a sensitivity of 71.9 % and a specificity of 68.9 %, MBG0/1 with a sensitivity of 72.6% and a specificity 68.6%, CTFC > 28 with a sensitivity of 76% and a specificity of 65.8% ( Figure 2 ).

**Table 2:** – Microvascular Perfusion Evaluated by Various Indices for Patients with FAR

	High FAR	Low FAR	p
**Myocardial Blush grade**			
MBG 0/1	44 ( 62 %)	18 (18.8 %)	<0.001
MBG 2/3	27 (38%)	78 (81.3)	<0.001
**Corrected TIMI frame count**			
>28	37 (52.1 %)	13 (13.5 %)	<0.001
≤28	34 (47.9 %)	83 (86.5 %)	<0.001
**ST-segment resolution**			
STR<50%	46 (64.8 %)	18 (18.8 %)	<0.001
STR≥50%	25 (35.2 %)	78 (81.3 %)	<0.001
**Enzymatic infarct size**			
Peak cTnT, pg/ml	5100 (3410-7890)	1670 (1022-4092)	<0.001
Peak CK-MB, U/L	255 (201-300)	133 (98-229)	<0.001

*FAR: Fibrinogen-Albumin Ratio; MBG: myocardial blush grade; STR: ST-segment resolution; cTnT: cardiac troponin T; CK-MB: creatine kinase-MB.*

**Table 3:** – Correlation between FAR and clinical, laboratory, and angiographic

Variables	FAR	
	r	p
NLR	0,389	<0,001
STR	-0,666	<0,001
Syntax Score	0,223	0,004
LVEF	-0,398	<0,001
Peak Troponın level	0,582	<0,001
CTFC	0,731	<0,001
MBG	-0,523	<0,001
Pain-to-balloon time (min)	0,158	0,041
LDL	0,245	<0,001
Glucose	0,387	<0,001

*FAR: Fibrinogen-Albumin Ratio; NLR: neutrophil/lymphocyte ratio; STR: ST-Segment Resolution; LVEF: left ventricular ejection fraction; CTFC: Correct TIMI frame count; MBG: myocardial blush grade; LDL: low-density lipoprotein.*

**Table 4:** – Logistic Regression Identifying Risk Factors for MBG 0/1, CTFC>28 and STR<50%

Variables	MBG 0/1		CTFC>28		STR<50%	
	Adjusted OR (95% CI)	p	Adjusted OR (95% CI)	p	Adjusted OR (95% CI)	p
FAR	0.219 (0.090-0.531)	<0.001	0.230(0.093-0.567)	<0.001	0.097 (0.037-0.250)	<0.001
Age	0.970 (0.931-1.10)	0.143	0.089(0.949-1.030)	0.586	1.006 (0.970-1.044)	0.750
DM	0.982(0.411-2.346)	0.967	1.448 (0.587-3.573)	0.422	0.867 (0.375-2.002)	0.738
SYNTAX	1.046 (0.978-1.119)	0.190	0.909 (0.854-0.967)	0.002	0.977 (0.925-1.032)	0.403
NLR	0.710(0.520-0.970)	0.031	0.989 (0.875-1.119)	0.864	0.929 (0.758-1.138)	0.476
Glucose	1.010 (0.989-1.022)	0.023	0.991 (0.982-1.001)	0.048	1.013(1.002-1.024)	0.018
LDL-C	1.007(0.988-1.027)	0.463	0.999 (0.981-1.017)	0.900	1.001(0.985-1.018)	0.881
Peak cTnT	1.010(.0997-1.028)	0.041	1.000 (0.988-1.012)	<0.001	1.001 (1.000-1.002)	<0.001
Pain-to-balon time	1.001(0.982-1.020)	0.025	1.035 (1.024-1.046)	0.002	0.977(0.960-0.994)	0.007

*FAR: Fibrinogen-Albumin Ratio; SYNTAX: SYNergy between percutaneous coronary interventin with TAXus; NLR: neutrophil/lymphocyte ratio; STR: ST-Segment Resolution; CTFC: Correct TIMI frame count; MBG: myocardial blush grade; LDL-C: low-density lipoprotein cholesterol.*

**Figure 2:** – *Receiver-operating characteristic curve analysis of the FAR levels for the prediction of incompleted ST-segment resolution (A), myocardial blush grade 0/1 (B), and correct TIMI frame count >28 (C). AUC: area under the curve; FAR: fibrinogen-to-albumin ratio.*

## Discussion

This study investigated the effect of FAR on microvascular reperfusion markers MBG, CTF, and STR. To our knowledge, this is the first prospective study conducted for this purpose in the literature. Our results showed that increased FAR was associated with low MBG, decreased STR, and high CTFC. It was also revealed that FAR was the most important independent factor in predicting these parameters.

STR after angiographically successful pPCI is closely related to tissue damage and perfusion, and many studies have shown that reduced STR is associated with poor clinical outcomes.^14 , 15^In the current study, it was also observed that all the patients with incomplete STR were in the high FAR group. It was also demonstrated that a high FAR level was associated with increased peak cTnT and low LVEF values, which are markers of tissue damage. Increased fibrinogen levels cause an increase in platelet activation and aggregation, resulting in a hypercoagulable state. In addition, the structure of a fibrin clot is closely related to the fibrinogen level. It has been reported that fibrin formed in vitro is more dense and more resistant to fibrinolysis at high fibrinogen concentrations than fibrin formed at low concentrations.^16^Albumin, the other component of FAR, is the major protein of human serum and inhibits platelet aggregation by increasing prostaglandin D2 production. In addition, a low albumin level is known to cause an increase in blood viscosity and deterioration in endothelial function. Furthermore, it is known that albumin has an antioxidant effect and an inverse relationship with inflammation.^7^In light of these mechanisms, we consider that a high FAR value may cause a decrease in STR by disrupting reperfusion at the microvascular level.

In recent studies, CTFC scoring has been used to evaluate reperfusion after PCI rather than the TIMI flow score since the latter offers a more objective and quantitative evaluation.^17 , 18^Many studies have shown that CTFC measurements provide useful results in predicting clinical outcomes. Accelerated coronary flow is known to be associated with good clinical outcomes. It has been shown that low CTFC after reperfusion is associated with a low mortality rate.^19^In the current study, we used CTFC to evaluate myocardial reperfusion provided by the artery responsible for myocardial infarction and determined that increased FAR was the most important predictor of CTFC > 28. It is considered that the responsible mechanisms in microvascular circulatory disorder are distal macroembolization, microembolization, local thrombus formation in the distal region, oxygen radicals released into the environment, increased calcium level in myocytes, interstitial edema, endothelial dysfunction, vasoconstriction, and inflammation.^20^Many studies have demonstrated the relationship of FAR with the abovementioned factors.^21^Similarly, in our study, impaired microvascular circulation in patients with high FAR may have resulted in a higher CTFC in this patient group.

Angiographic success after PCI is defined as at least a 50% reduction in stenosis with the achievement of grade 3 TIMI flow after balloon dilatation and a maximum of 10% residual stenosis after stenting.^12^However, successful angiographic outcomes do not always mean successful myocardial reperfusion. Due to its practicality and ease of use, MBG is frequently used in clinical practice to determine microvascular perfusion. MBG 0/1 is defined as a perfusion disorder at the microvascular level.^13^A recent meta-analysis of 8,044 patients showed that MBG 0/1 after primary angioplasty was associated with all-cause mortality.^22^In the current study, we determined that increased FAR was an important predictor factor for MBG 0/1. The increased thrombogenic effect of fibrinogen alone and the low albumin level increase platelet aggregation, leading to perfusion disorder at the tissue level. In addition, FAR is considered to be related to increased blood viscosity and thrombogenicity.^7^It has been suggested that the increased thrombus load of these factors impairs myocardial perfusion by causing distal microembolism.^23 , 25^Parallel to these mechanisms, a high FAR value was associated with MBG 0/1 in our study. Another important finding of our study is that the patients in the high FAR group had a higher SYNTAX score, which is consistent with the results reported by Erdoğan et al.^26^İn our study, we also found that glucose, LDL-C levels, and NLR were significantly higher in the high FAR group than the low FAR group. Recently, FAR has been reported as an inflammation marker related to various diseases, including diabetes mellitus and hyperlipidemia.^27 , 28^

### Limitations

There were some limitations to this study due to its design. First, the study population was relatively small. Second, we measured only at the admission, and it was not evaluated after the acute phase can be considered an important limitation. Third, this parameter’s lack of effect on cardiovascular outcomes, including re-intervention and mortality.

## Conclusions

In conclusion, based on our results, we consider that FAR is an easily accessible and inexpensive marker that can be used by clinicians in the evaluation of microvascular perfusion in patients that have undergone pPCI for STEMI. In addition, this marker can be used to determine adjuvant treatment options for pPCI.
